# The Campaign against Malaria

**Published:** 1908-10-10

**Authors:** 


The Hospital

A Journal of

The Medical Sciences and Hospital Administration.

New Series. No. 83, Vol. IV. [No. 1155, Vol. XLV.] SATURDAY, OCTOBER 10, 1908.

### THE CAMPAIGN AGAINST MALARIA.

There has been published recently a report upon the prevalence and prevention of malaria in Mauritius. The report is from the pen of Major Ronald Ross, who, at the request of the Colonial Office, spent three months on the island in obtaining first-hand information. It appears that until the middle of the last century Mauritius was tolerably free from malaria; at all events, the disease was not then endemic as it is to-day. Between 1858 and 1867 there occurred several epidemic outbreaks, and in the last of these years the island was visited by a disastrous onslaught of malaria which destroyed one fifth of the total population. In Port Louis there died on one day, April 27, 1867, 234 people out of a population of 87,000; while during the month more than 6,000 souls perished from malaria and causes incidental to the pestilence. In the whole island nearly 32,000 deaths were ascribed to fever during the year 1867, the population at the time being estimated at 360,000 souls. Ever since this great outbreak the disease has been endemic, the fever death-rate amounting to 31 per cent. of the total in the period between 1900 and 1906. The extent of this endemicity may be gauged from the fact that of 31,000 children no less than one-third were found to be the subjects of chronic enlargement of the spleen, that is to say, capable of supplying the infective agent so long as the appropriate mosquitoes are present to avail themselves of the supply. The medical officer of the Emigration Department has estimated that the annual cost of the disease to the sugar estates, which represent the principal industry of the island, is 650,000 rupees in loss of labour, while the annual loss of wages to the labourers themselves amounts to R. 150,000.

It is clear then that the call for an attack upon malaria in Mauritius is urgent, and the plain man may be forgiven for wondering why the call has been so long disregarded. As Major Ross observes, the cause and mode of prevention of the most widespread and important tropical disease has been discovered and taught, yet the advantage taken of this knowledge has so far been quite disproportionate to the value of it. Here and there in limited areas, ague-stricken districts of enduring notoriety have been robbed of their evil eminence, but progress is unintelligibly slow. The most striking example of what can be done in this respect under a determined and capable administration is the case of the isthmus of Panama. Under the French *régime* the attempt to cut the canal was foiled principally by the malaria-bearing anopheles, and the stegomyia of yellow fever. The day was with these pests, and the cutting was given up, the effort having cost some 50,000 lives. With the advent of the Americans the scheme of attack was totally recast, and the first care of those in authority was to solve the sanitary problem. Colonel Gorgas was put in command of the sanitary campaign, the objective of which was the abolition of mosquitoes by the destruction of their breeding-grounds in swampy areas. How well the task was done is shown by the mortality returns. During 1906 the death-rate among 5,000 white American workmen was 7 per 1,000, the death-rate from disease amounting to no more than 3.8 per 1,000.

It is no wonder that, with such potentialities in his mind, Major Ross urges a formal and comprehensive attack upon the problem before Mauritius. There are two main items in the business—first the elimination of the parasite, and second, the elimination of the mosquitoes which distribute it. As regards the first of these items, it is clear that every case of chronic malaria which is cured means one source of parasites the less. It is, therefore, of the highest moment that chronic cases of malaria should be kept under observation and cured if possible, instead of continuing to be a perpetual fountain of infection. For this purpose, Major Ross recommends periodical examination of school children for splenic enlargement, active treatment of the disease wherever found, and continuous house-to-house distribution of quinine. In addition he gives, under the heading "Personal Prevention," practical hints upon the means of avoiding infection if circumstances entail a sojourn in the near neighbourhood of an infected area. There is nothing new in this advice, nor is anything required beyond it. Yet the hardest task before apostles of hygiene is to secure the attention of the multitude for simple personal precautions, the wisdom of which would seem to be so obvious as to need no recommendation. Ignorance and carelessness are the two prime enemies of hygienic progress, and these can only be overcome painfully and by much perseverance. Even among educated English people, who have lived long in tropical climates, one hears "fever" spoken of with a contemptuous familiarity which does not augur well for enthusiasm in the fight against it. It is no wonder then that ignorant coloured populations should incline to accept fever with a fatalistic resignation. It is in the drainage of marshes and the destruction of the stagnant pools in which the larval stage of the mosquito is passed that public prevention finds its directest route to success. Such drainage schemes, of course, are costly, and this probably accounts for the delay in instituting them. It has often been observed that mankind will pour out money to remedy a mischief which might have been avoided altogether by the timely expenditure of a tithe of the sum so lavishly supplied too late. Major Ross estimates that the carrying out of his recommendations will cost Mauritius 135,000 rupees annually. We have seen that the estimated annual loss to the island resulting from malaria is 800,000 rupees. The balance seems worth saving even on its own account, and apart from all considerations of humanity.

